# mGem: More than building blocks? Mitochondrial metabolism, viruses, and the host response to infection

**DOI:** 10.1128/mbio.02501-25

**Published:** 2026-05-05

**Authors:** Jessica Alvarez, Dustin C. Hancks

**Affiliations:** 1 Molecular Microbiology Ph.D. program, UT Southwestern Medical Center 12334 , Dallas, Texas, USA; 2 Department of Immunology, UT Southwestern Medical Center 12334 , Dallas, Texas, USA; The Ohio State University, Columbus, Ohio, USA

**Keywords:** mitochondria, immunometabolism, viral infection, immune response, OXPHOS, mitochondrial dysfunction, sterile inflammation

## Abstract

Beyond essential roles as central hubs integrating homeostatic cellular metabolism, mitochondria have emerged as critical determinants of infection outcomes. Mitochondrial activities, like MAVS signaling and the release of cytochrome c and mitochondrial DNA, drive host defenses. Across cell types, mitochondrial metabolism and antiviral responses are also increasingly being connected by evidence such as viral-encoded antagonists. Nonetheless, metabolic rewiring in infected cells is still largely viewed as a means to satisfy biosynthetic demands for both viral replication and the host response. However, perturbation of metabolic states within infected and bystander cells seemingly has consequences for outcomes, implying an incompletely understood metabo-immunoregulatory logic. Here, we consider roles for mitochondrial metabolism reprogramming as an active cue that licenses progressive immune states to adapt host responses. In the coming years, integration of mitochondrial biology and new methodologies, including spatial approaches, will illuminate the interplay of mitochondrial metabolism on primary antiviral responses.

## PERSPECTIVE

Co-option of mitochondria is generally accepted as a consequence of endosymbiosis of an ancestral α-proteobacterium due to a perceived metabolic advantage provided by bioenergetic and biosynthetic activities (1). From studies largely with mammalian systems, it is also clear that mitochondria act as vital and often early architects for the host response (2–4) to pathogens. Now well-appreciated, mitochondria transduce different signals and release damage-associated molecular patterns (DAMPs) to produce type I interferons: retinoic acid-inducible gene I (RIG-I)-like receptor/mitochondrial antiviral signaling (MAVS) pathway (5), mitochondrial DNA (mtDNA) release (6), and mitochondrial RNA (mtRNA) release (7). Notably, mitochondria advance the apoptotic cell death program by releasing the electron transport chain (ETC) carrier protein cytochrome c into the cytosol (8). Although these defenses are critical facets of the host arsenal, they are not a major focus here and have been previously reviewed (9–11). For this mGems review, we consider the consequences of metabolic states in infected cells and their impact on outcomes.

An emanating perspective points to mitochondrial reprogramming as a conserved and genetically encoded adaptation to counter viral replication. While still viewed by many as non-overlapping classes of biological activities, metabolism and immunity have a rich history of crosstalk (12) and may be even more deeply intertwined than previously appreciated (4). A quintessential example is the master metabolic regulator, mechanistic target of rapamycin (mTOR) (13), which was discovered because of work on the immunomodulatory properties of rapamycin (14, 15). Interestingly, mTOR now has demonstrated roles in cytosolic sensing and antiviral responses, which were uncovered from studies of a conserved poxvirus protein that directly targets the mTOR complex (16–18). Conversely, tumor necrosis factor-alpha (TNF-α)—a master regulator of inflammation—has several roles in metabolic regulation (19, 20), including the modulation of insulin resistance, which is a signature of metabolic disease (21). Perhaps viral-encoded antagonists for TNF-α signaling, such as poxvirus decoy receptors (22, 23), perturb these functions.

**Fig 1:**
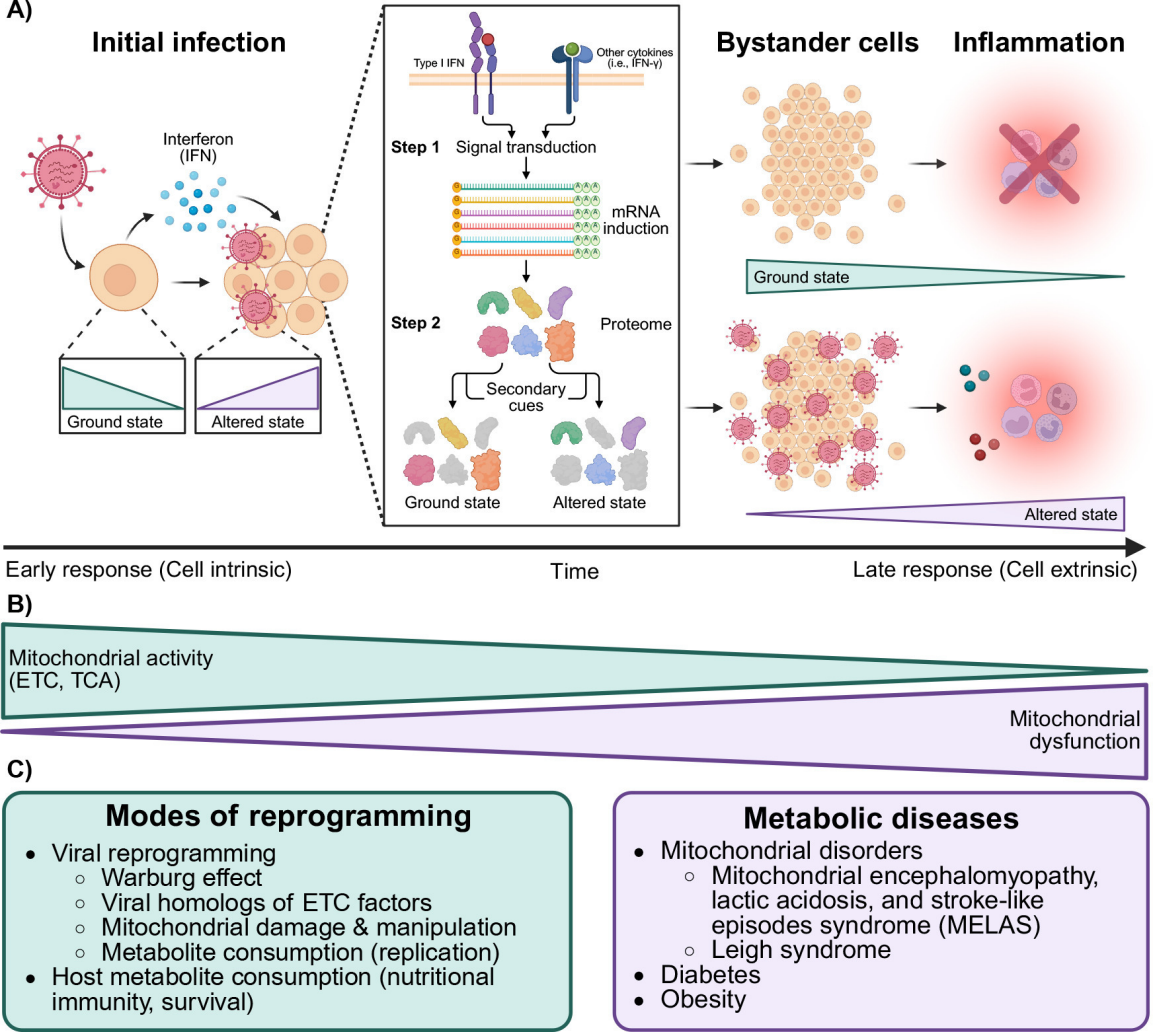
Emerging model of mitochondrial activity during viral infection. (**A**) Host responses to infection progress from innate defenses in initially infected cells to activated responses in bystander cells to late-stage cell-extrinsic immunity mediated by inflammation. Cell-intrinsic responses, including interferon (IFN) stimulated defenses, are deployed early in infection. Proposed model consisting of a two-step reprogramming cascade involving host defenses and metabolic states. Step 1: immune signal transduction leading to mRNA induction. Step 2: adaptation either by augmentation or altered composition of the host response, including induced immune factors, mediated by metabolic state; ground state of homeostatic metabolism and OXPHOS vs. altered state of mitochondrial dysfunction and reliance on other metabolic programs. (**B**) This model suggests that perturbation of the metabolic state during infection adapts the host response from cell-intrinsic defenses to advanced responses like inflammation. (**C**) Viruses may exploit cellular metabolism through multiple reprogramming mechanisms: aerobic glycolysis (Warburg effect) (34), viral homologs of ETC factors (35), and direct mitochondrial damage (36). Both the host and viral response can cause differences in metabolite availability. Informative parallels may exist between infected cells and diseases with mitochondrial dysfunction, such as in mitochondrial diseases, which display aberrant inflammatory responses, including mitochondrial encephalomyopathy, lactic acidosis, stroke-like episode syndrome (MELAS), and Leigh syndrome (37, 38).

Over the last 15 years, there has been an explosion of research exploring how metabolic changes influence effector functions of immune cells (24–26) (e.g., macrophages, T cells). This research area is termed immunometabolism (27). Metabolic changes in infected cells (28, 29), many of which are also non-immune cells (30, 31), are well-documented (32, 33). In virology, reprogramming of metabolism and mitochondrial activity is typically perceived as both (i) a means to satisfy biosynthetic and bioenergetic needs for pathogen replication and (ii) a hallmark of disrupted cellular homeostasis. Notwithstanding, mitochondrial reprogramming is also a nearly universal feature of the host response during infection (Fig. 1). Consequently, it should be considered in light of the selective pressure imposed by pathogens, which have likely shaped this activity and its effects. Such framing may inform the biology of sterile inflammation, which is associated with a myriad of complex diseases and co-occurs with mitochondrial dysfunction. Hereafter, we discuss interactions between viruses and mitochondrial metabolism, and how it may be more than a strategy to obtain biological building blocks.

## MAYBE LESS A GROCERY STORE AND MORE A CANARY IN THE COAL MINE

Mitochondria have established housekeeping functions (10, 39) that are used to signal distress. The seminal finding bridging mitochondrial metabolism to cell-intrinsic immunity (8) was the discovery that cytochrome c released into the cytosol by mitochondrial outer membrane permeabilization catalyzed apoptosome formation. To no surprise, mitochondrial damage-associated molecular patterns (DAMPs) are released by various types of insults. These include (i) mitochondrial permeabilization by host (40) or viral proteins (36), (ii) stress (41–43), and even (iii) downregulation of mitochondrial biogenesis (44). Damage to the organelle is not always absolute (45) and is mitigated, in part, by homeostatic processes that overlap with host defenses such as mitochondrial recycling via mitophagy (46), biogenesis (47), and mitochondrial fusion and fission (48).

Mitochondrial perturbation is commonly associated with downregulation of ETC activity. Decreased ETC activity leads to reliance on other metabolic states like glycolysis (49, 50). Upregulation of glycolysis in macrophages and other immune cells is linked to pro-inflammatory activities like cytokine production (51). Notably, this mitochondrial rewiring also seems to apply to non-immune cells. For example, upregulated glycolysis and pro-glycolytic conditions in nasal epithelial cells (52), uterine cells (53), and keratinocytes (54) also result in increased pro-inflammatory cytokine expression (e.g., IL1, TNF-α, CXCL8, and CCL20). Relatedly, other key inflammatory factors such as cyclooxygenase-2 (COX2) and interferon regulatory factor 1 (IRF1) are increased by pro-glycolytic conditions in immune cells (55) and non-immune cells (56–58). Thus, shifting from homeostatic mitochondrial metabolism to glycolysis as a consequence of mitochondrial perturbation may be a universal cue to inherently poise cells to favor inflammation and prime them for an advanced stage response (24, 59, 60).

## EQUAL AND OPPOSITE FORCE: VIRAL PROTEINS TARGETING MITOCHONDRIA AND METABOLIC FUNCTIONS

Regulation by antiviral signals may implicate a specific host metabolic factor in infection outcomes. As viruses counteract host activities that are detrimental to viral fitness, identification of virus-encoded antagonists of host metabolic factors provides additional evidence of their relevance in infection. Several metabolic changes during the host response and infected cells, including shifts in the production of amino acids (61, 62), lipids (63–65), nucleotides (66, 67), electron transport chain activity (68–70), and polyamines (71), have been linked to cellular effectors. Particularly interesting are infection-induced lipid droplets that impart an antiviral state (72). Given that a subset of these host metabolic factors are interferon-stimulated genes (ISGs) (Table 1), this further indicates that some of the observed metabolic alterations indeed promote antiviral states rather than merely representing indirect consequences of infection (35, 73).

**TABLE 1:** Examples of viral and host response proteins associated with metabolic reprogramming^*a*^

Factor	Effect
Virus (factor)	Mitochondrial
ASFV (MGF360-16R)	↑ apoptosis (74)
DENV (NS3)	↑ FAS (75, 76)
EBV (BHRF1)	↓ apoptosis (77, 78)
EV-D68 (VP3)	↓ NF-kB activation (79)
HBV (HBx)	↓ glycolysis, ↑ mitophagy (80, 81)
HCMV (UL13)	↑OXPHOS (82)
HCMV (vMIA/UL37 x 1)	↑ lipid metabolism, ↓ apoptosis (83–85)
HCV (Core)	↑ lipid metabolism, ↑ ROS (86, 87)
HIV (Vpr)	↑ ROS, ↑ apoptosis (88, 89)
HPV-16 (E1^E4)	↑ apoptosis (90)
HPV-18 (E2)	↑ glycolysis, ↑ ROS (91)
HSV-1 (UL12.5)	mtDNA damage (92)
HTLV-1 (p13)	↑ apoptosis (93)
IAV (PB1-F2)	↑ mitochondrial fragmentation (94)
JEV (NS4A)	↑ mitophagy (95)
JEV (NS5)	↓ β-oxidation (96)
KSHV (vBcl-2)	↑ mitochondrial fission (97)
MNV (NS3)	↑ mitochondrial fragmentation, ↑ ROS (36)
MYXV (M11L)	↓ apoptosis (98)
PEDV (Nsp14)	↑ mitophagy (99)
SARS-CoV-2 (ORF10)	↑ ROS, ↓ mitophagy, ↓ amino acid metabolism (100, 101)
SQPV (vMISTRAV/SQPV078)	↓ apoptosis (35)
VACV (D10)	mRNA metabolism (102)
VACV (F1L)	↓ apoptosis (103)
VACV (F17)	↓ glycolysis (18)
ZIKV (NS4A)	↑ ROS, ↑ mitophagy (104)
ZIKV (NS4B)	↑ apoptosis (105)
Host factor	Mitochondrial
CMPK2	↓ nucleotide metabolism (106)
IFI27	↑ apoptosis (107)
IRG1	↓ glycolysis (108)
MCJ	↓ OXPHOS (69)
MISTR1/NDUFA4	ETC interacting factor (35)
MISTRAV/C15orf48	ETC interacting factor (35)
MISTRH/NDUFA4L2	↓ OXPHOS, ↑ glycolysis (36, 109)
PDK1	↑OXPHOS (110)
PNPT1	dsRNA release (7)
SDH	↑ ROS (111)
Host factor	Non-mitochondrial
ARG1/2	Changes in nutrient availability (112)
CH25H	Changes in cholesterol metabolism (113, 114)
ENPP1	Nucleotide metabolism (115)
IDO1	Changes in nutrient availability (62)
ISG15	Lipid metabolism, ↓ glycolysis (116, 117)
PFKFB3	↑ glycolysis (118)
SAMHD1	Changes in nutrient availability (66)
SIRT3	↓ glycolysis (119)
Viperin	↓ ribonucleotide synthesis (120)

^*a*^ Viral proteins are organized by virus of origin with the specific viral factor shown in parentheses. Host factors were retrieved from the literature or lists of known ISGs (121) (interferon-stimulated genes). Effects on mitochondrial processes are indicated with arrows (↑ = increase/promotion; ↓ = decrease/inhibition). Abbreviations: ASFV, African swine fever virus; DENV, dengue virus; EBV, Epstein-Barr virus; EV-D68, enterovirus D68; HBV, hepatitis B virus; HCMV, human cytomegalovirus; HCV, hepatitis C virus; HIV, human immunodeficiency virus; HPV, human papillomavirus; HSV-1, herpes simplex virus type 1; HTLV-1, human T-cell lymphotropic virus type 1; IAV, influenza A virus; JEV, Japanese encephalitis virus; KSHV, Kaposi’s sarcoma-associated herpesvirus; MNV, murine norovirus; MYXV, myxoma virus; PEDV, porcine epidemic diarrhea virus; SARS-CoV-2, severe acute respiratory syndrome coronavirus 2; SQPV, squirrelpox virus; VACV, vaccinia virus; ZIKV, Zika virus; ETC, electron transport chain; mtDNA, mitochondrial DNA; NF-κB, nuclear factor kappa B; OXPHOS, oxidative phosphorylation; ROS, reactive oxygen species.

Interpretations of antiviral activity mediated by metabolism are complicated. Discerning the relevant consequences of metabolic reprogramming on viral replication remains challenging, in part, due to crosstalk with immune defenses. Specifically, metabolic reprogramming could limit viral replication by either starving the virus, similar to nutritional immunity against bacteria (122) and parasites (123), thereby creating a toxic state through redox stress, or through other more direct antiviral effects. An example of a direct antiviral “metabolic factor” is the ISG viperin, which produces chain-terminating ribonucleotides to inhibit viral replication (120). Moreover, certain changes in metabolism like nutrient restriction trigger stress responses that represent pivotal antiviral responses (i.e., the integrated stress response [124]). Extending this concept, other major shifts in metabolism, including flux (125) or modulation of ETC respiration (126), might also be sensed as altered self and serve to adapt defenses.

Many viruses and viral proteins (Table 1) antagonize mitochondrial and metabolic machinery. Proviral aspects of metabolic reprogramming are supported by the identification of specific metabolites that favor viral replication, such as glutamine over glucose (127). Supporting the notion that viruses must manipulate mitochondrial metabolism to establish productive infection, diverse viruses target core metabolic pathways that interface with mitochondria, including the ETC (35, 82), tricarboxylic acid cycle (TCA) cycle, reactive oxygen species (ROS) (128), nicotinamide adenine dinucleotide (NAD(H)) (129) metabolism, and fatty acid metabolism (29). In some cases, the viral-encoded antagonists are derived from cellular genes such as ETC subunits (35) or near-complete sets of TCA cycle enzymes (130). Indirectly, many viral proteins also disrupt mitochondrial metabolism by damaging or permeabilizing mitochondria. Viral-mediated turnover, damage, and modulation of mitochondrial networks is prevalent, but the advantage in many cases is incompletely understood. Remodeling mitochondrial networks and abundance could function as a strategy to deplete mitochondrial-associated host defense effector molecules whether it be ROS, MAVS, cytochrome c, or mtDNA. In principle, viral disruption of mitochondrial metabolism could be a scheme to promote aerobic glycolysis and increase the levels of lactate to directly inhibit MAVS (131). Altogether, virus counteraction of host- and/or stress-mediated metabolic reprogramming may represent ill-defined approaches to suppress host defenses either by exploiting regulatory circuitry or mimicking the aspects of homeostasis.

## WHY TIP THE BOAT? MITOCHONDRIAL METABOLIC REPROGRAMMING AS A DAMP

Given the nuanced diversity of intracellular pathogens, the cell types and the hosts they infect, it seems improbable that metabolic reprogramming is solely a mechanism for viral sustenance. As viruses exploit cellular circuitry, nutritional immunity in this context inherently decreases host fitness. Notably, there appears to be an innate directionality to the host responses associated with metabolic reprogramming. Specifically, progression is largely constrained to move from mitochondria to other metabolic programs like glycolysis in both infected cells and disease states. In both cases, the host response progresses to an advanced immune stage, namely inflammation.

Mitochondria have been proposed to function as sensors (132) in the host response. The extensive involvement of mitochondria at various levels of immunity is attributed to a break in endosymbiosis (4), which supports the idea that these ancient bacteria have come to a truce. Besides MAVS, more ancestral features of mitochondria, particularly in ground metabolic states like normal respiration, may enable sensing of altered self. Disruption of homeostatic metabolism may be mediated by (i) mitochondrial permeabilization as a consequence of viral egress (36), (ii) expression of host (e.g., ISGs) and viral proteins with metabolic activities (Table 1), and (iii) consumption of cellular metabolite pools as a consequence of viral replication (56).

A potential fourth way to disrupt mitochondrial metabolism is the release of mtDNA into the cytosol, which commonly occurs as a result of viral infection. In cultured cells, depletion of mtDNA in mitochondria over time abolishes oxidative phosphorylation (OXPHOS) (133, 134). This is because the levels of ETC complexes are downregulated due, in part, to decreased expression of mtDNA-encoded ETC subunits. Thus, host responses induced by sensing of cytosolic mtDNA mediated by pattern recognition receptors, such as cyclic GMP-AMP synthase (cGAS) (6), NLR family pyrin domain containing 3 (NLRP3) (135, 136), and absent in melanoma 2 (AIM2) (137), might be augmented by OXPHOS dysfunction. If this is the case, the principle may also apply to conditions where mtRNA is released into the cytosol. In addition, rewiring of metabolic state either by culturing conditions (56) or targeting of metabolic effectors (138, 139) is sufficient to change the composition of the host response and tip infection outcomes by altering the levels of select antiviral factors, IRF1 and IFITM3. Thus, several lines of evidence support mitochondrial metabolic activity may instruct the stage of infection.

Based on the points raised herein and integrating insights from virology, immunometabolism, and metabolic diseases, we propose a two-step model for the functional interplay of antiviral host defenses and metabolic states (Fig. 1A). The first step involves the initial viral sensing, which induces innate immune signaling including ISG mRNA expression. In the second step, virus-driven or host-driven metabolic rewiring acts as an instructive signal to rapidly adapt the composition of the response to cue the stage of infection, either early or advanced. Therefore, functional mitochondrial metabolism promotes the early, cell-intrinsic response, whereas mitochondrial dysfunction signals the advanced response of inflammation. Altogether, this relationship influences which antiviral effectors are ultimately produced and thereby shapes infection outcomes.

## EMERGING TOOLS AND CONSIDERATIONS

Studying mitochondrial functions during the host response involves several considerations. These include the infection kinetics, as mitochondrial phenotypes can shift dramatically across the infection time course and cell type. Likewise, most cells are grown in a glucose-rich medium, which does not mirror the metabolic state encountered by the virus *in vivo* (i.e., OXPHOS). In infected and bystander cells, early metabolic changes may reflect host defense responses, while late-stage alterations may indicate viral manipulation or accumulating cellular stress. The importance of timing and metabolic state is illustrated by extended interferon priming inhibiting poxvirus replication by stabilizing the inflammatory transcription factor IRF1 in glycolytic but not OXPHOS-promoting conditions (56). These data suggest that sustained bioenergetic state influences the durability of antiviral programs beyond initial gene induction.

Tracking of mitochondrial dynamics (140, 141) using live-cell mitochondrial reporters, mito-Keima for mitophagy and mito-TIMER for mitochondrial age, with reporter viruses will likely lead to new insights. To resolve cellular heterogeneity in infection models, fluorescent labeling with fluorescence-activated cell sorting and mass spectrometry can isolate cells at different distances from infection, potentially revealing differences in mitochondrial activity within microenvironments (142). Spatial transcriptomics platforms may inform the relationship between mitochondrial and metabolic gene expression shifts across different microenvironments within infected tissues. Likewise, leveraging reporter viruses with stage-specific promoters will complement data integration for the immune response, including ISG induction, with metabolic states in relation to infected cells. These analyses could reveal whether cells at increasing distances from the infection undergo progressive metabolic shifts preceding, concurrent, or subsequent to immune signaling. These technologies hold promise for elucidating new insights into mitochondrial biology.

## SUMMARY

Mitochondrial dysfunction concomitant with inflammation in infection and many human diseases suggests that sensing of altered metabolic state itself may have evolved to function as a vital secondary cue to adapt host immunity. Emerging tools enabling spatial and temporal resolution of mitochondrial biology in infected cells, combined with insights from diseases and models characterized by mitochondrial dysfunction (143–145), promise to clarify these relationships and reveal new therapeutic opportunities.
